# Supplementary material for: Mechanisms governing the pioneering and redistribution capabilities of the non-classical pioneer PU.1
Source: Nat Commun. 2020 Jan 21;11:402. doi: 10.1038/s41467-019-13960-2 (PMC6972792; doi:10.1038/s41467-019-13960-2)
Supplement: Supplementary file 7 — Source data [file 41467_2019_13960_MOESM7_ESM.zip › Source_Data/FigureS2/FigureS2B_MotifScanOutput/KmeansCluster10/homerResults.html]

/misc/data/analysis/project\_PU1/CTV1/motifs/basic/PU1\_Kmeans14.10/final - Homer de novo Motif Results


# Homer *de novo* Motif Results (/misc/data/analysis/project\_PU1/CTV1/motifs/basic/PU1\_Kmeans14.10/final)

Known Motif Enrichment Results  
Gene Ontology Enrichment Results  
If Homer is having trouble matching a motif to a known motif, try copy/pasting the matrix file into
STAMP  
More information on motif finding results: HOMER
| Description of Results
| Tips
  
Total target sequences = 3712  
Total background sequences = 44191  
\* - possible false positive  

|  |  |  |  |  |  |  |  |  |
| --- | --- | --- | --- | --- | --- | --- | --- | --- |
| Rank | Motif | P-value | log P-pvalue | % of Targets | % of Background | STD(Bg STD) | Best Match/Details | Motif File |
| 1 | A T G C C T G A A T G C G C A T C G A T A T G C A G T C G A C T A G T C G C A T G A C T G A C T | 1e-1669 | -3.845e+03 | 77.18% | 11.08% | 30.6bp (72.8bp) | PB0058.1\_Sfpi1\_1/Jaspar(0.948) More Information \| Similar Motifs Found | motif file (matrix) |
| 2 | A G T C A G T C A C G T A G T C A T G C A G C T A G T C | 1e-334 | -7.700e+02 | 64.25% | 31.51% | 53.5bp (71.1bp) | SRSF1(RRM)/Homo\_sapiens-RNCMPT00163-PBM/HughesRNA(0.839) More Information \| Similar Motifs Found | motif file (matrix) |
| 3 | T C G A T C G A T C G A C G T A T C A G A G T C C T G A C T A G G C T A G C T A T C A G C A G T T A C G | 1e-130 | -3.003e+02 | 9.46% | 1.58% | 37.4bp (69.4bp) | SpiB(ETS)/OCILY3-SPIB-ChIP-Seq(GSE56857)/Homer(0.881) More Information \| Similar Motifs Found | motif file (matrix) |
| 4 | A T C G G A C T A G C T A T G C A T G C A C G T C A T G | 1e-105 | -2.427e+02 | 43.43% | 26.05% | 53.9bp (69.1bp) | ZNF638(RRM)/Homo\_sapiens-RNCMPT00164-PBM/HughesRNA(0.767) More Information \| Similar Motifs Found | motif file (matrix) |
| 5 | C A T G G A T C A G T C C T G A T A G C G A C T C T G A A C T G C T G A A C T G A C T G C A T G G A T C T C G A | 1e-101 | -2.337e+02 | 4.47% | 0.31% | 52.7bp (54.8bp) | CTCF(Zf)/CD4+-CTCF-ChIP-Seq(Barski\_et\_al.)/Homer(0.927) More Information \| Similar Motifs Found | motif file (matrix) |
| 6 | A T C G A G C T C G A T T G A C A T G C A G C T A G T C T G A C A G T C G T A C | 1e-93 | -2.148e+02 | 20.99% | 9.20% | 50.4bp (73.0bp) | NCU02404(RRM)/Neurospora\_crassa-RNCMPT00238-PBM/HughesRNA(0.794) More Information \| Similar Motifs Found | motif file (matrix) |
| 7 | T A G C G T C A G A T C C G A T G C A T A T G C A G T C G C A T T C A G A G T C G T A C | 1e-93 | -2.143e+02 | 9.35% | 2.24% | 58.8bp (68.7bp) | ETS1(ETS)/Jurkat-ETS1-ChIP-Seq(GSE17954)/Homer(0.760) More Information \| Similar Motifs Found | motif file (matrix) |
| 8 | G C T A C G T A T C G A A G T C A G T C C G T A T A G C G C T A C G A T | 1e-89 | -2.066e+02 | 23.95% | 11.50% | 52.8bp (64.3bp) | RUNX1(Runt)/Jurkat-RUNX1-ChIP-Seq(GSE29180)/Homer(0.963) More Information \| Similar Motifs Found | motif file (matrix) |
| 9 | C A T G A C G T A C G T A T G C G A T C G A C T A T G C G A C T A C G T G A C T C A G T A C T G | 1e-87 | -2.019e+02 | 7.35% | 1.45% | 44.7bp (70.8bp) | PI/MA0559.1/Jaspar(0.799) More Information \| Similar Motifs Found | motif file (matrix) |
| 10 | C T A G T C A G G C T A G T C A A C T G A C G T A C T G A C T G A G C T A C G T C G T A | 1e-80 | -1.846e+02 | 2.91% | 0.12% | 56.6bp (59.6bp) | ETS:RUNX(ETS,Runt)/Jurkat-RUNX1-ChIP-Seq(GSE17954)/Homer(0.781) More Information \| Similar Motifs Found | motif file (matrix) |
| 11 | A T G C G C T A A T G C A C G T C A G T A G T C G A T C A G C T G A T C C G T A T C G A C G T A | 1e-66 | -1.539e+02 | 5.71% | 1.15% | 41.4bp (63.3bp) | PU.1(ETS)/ThioMac-PU.1-ChIP-Seq(GSE21512)/Homer(0.718) More Information \| Similar Motifs Found | motif file (matrix) |
| 12 | A C T G G T C A A C T G A C T G C G T A C G T A A T G C C T G A C T A G | 1e-65 | -1.497e+02 | 10.88% | 3.91% | 44.3bp (72.0bp) | ZNF189(Zf)/HEK293-ZNF189.GFP-ChIP-Seq(GSE58341)/Homer(0.773) More Information \| Similar Motifs Found | motif file (matrix) |
| 13 | T A G C A G T C C G T A A G T C C G A T G C A T A T G C A G T C A G T C C G A T C G T A | 1e-62 | -1.444e+02 | 3.74% | 0.47% | 46.1bp (54.4bp) | SD0002.1\_at\_AC\_acceptor/Jaspar(0.733) More Information \| Similar Motifs Found | motif file (matrix) |
| 14 | C T G A C G T A G T C A A T G C T A G C C T A G A T C G T G C A T C G A A T G C | 1e-61 | -1.406e+02 | 12.07% | 4.81% | 54.6bp (66.1bp) | prd/dmmpmm(Down)/fly(0.734) More Information \| Similar Motifs Found | motif file (matrix) |
| 15 | T G A C A C T G A C T G C G T A C T G A G T A C A G C T G T C A | 1e-60 | -1.384e+02 | 25.22% | 14.44% | 53.4bp (67.8bp) | ERT1/MA0420.1/Jaspar(0.761) More Information \| Similar Motifs Found | motif file (matrix) |
| 16 | T A G C T G C A T G A C C T A G A T C G C T G A C T G A A T C G C G A T A C T G A C T G | 1e-47 | -1.091e+02 | 3.96% | 0.78% | 47.7bp (64.1bp) | Gabpa/MA0062.2/Jaspar(0.808) More Information \| Similar Motifs Found | motif file (matrix) |
| 17 | C A T G A T C G G A T C C G T A A C T G A G C T A C G T A G T C G A T C T G A C G C A T A G C T | 1e-43 | -1.007e+02 | 4.23% | 0.97% | 53.6bp (65.7bp) | dl/dmmpmm(Papatsenko)/fly(0.679) More Information \| Similar Motifs Found | motif file (matrix) |
| 18 | T G C A T G C A G T A C G T A C G T C A G T A C G T C A G C A T A T G C G A T C A G C T A T G C G A C T | 1e-39 | -8.994e+01 | 3.74% | 0.85% | 44.8bp (66.8bp) | ETS:RUNX(ETS,Runt)/Jurkat-RUNX1-ChIP-Seq(GSE17954)/Homer(0.882) More Information \| Similar Motifs Found | motif file (matrix) |
| 19 | G A T C G T C A C T A G T C G A A C G T T C A G T C A G A G T C C T A G | 1e-35 | -8.149e+01 | 5.93% | 2.11% | 54.9bp (61.6bp) | CTCF/MA0531.1/Jaspar(0.852) More Information \| Similar Motifs Found | motif file (matrix) |
| 20 | C G T A T G C A A C G T A G T C C T A G A T C G G T A C C G T A | 1e-33 | -7.706e+01 | 29.58% | 20.75% | 55.9bp (65.7bp) | ETV2/MA0762.1/Jaspar(0.692) More Information \| Similar Motifs Found | motif file (matrix) |
| 21 | A C G T C T A G A T C G A G C T A C G T A G C T A G T C A G T C G C A T G A T C G C T A C G A T C G A T | 1e-30 | -7.036e+01 | 2.34% | 0.42% | 44.5bp (60.7bp) | GCR1/Literature(Harbison)/Yeast(0.734) More Information \| Similar Motifs Found | motif file (matrix) |
| 22 | C T A G A G T C A G T C C T G A A G T C C T A G T C G A | 1e-30 | -6.932e+01 | 25.84% | 17.89% | 56.4bp (61.6bp) | MET32/MA0334.1/Jaspar(0.866) More Information \| Similar Motifs Found | motif file (matrix) |
| 23 | C A T G A G T C C G A T A C T G A G T C T G A C T G C A | 1e-28 | -6.473e+01 | 37.10% | 28.30% | 57.1bp (63.8bp) | NF1-halfsite(CTF)/LNCaP-NF1-ChIP-Seq(Unpublished)/Homer(0.801) More Information \| Similar Motifs Found | motif file (matrix) |
| 24 | T G C A A C G T C T A G C G T A A C T G C A G T G A T C G C T A A C G T A G T C G C A T | 1e-27 | -6.256e+01 | 7.76% | 3.70% | 55.0bp (64.0bp) | JunB(bZIP)/DendriticCells-Junb-ChIP-Seq(GSE36099)/Homer(0.961) More Information \| Similar Motifs Found | motif file (matrix) |
| 25 | T G C A T G A C T G A C G C T A T G A C G C T A C A G T G A T C T C G A T G C A C T G A A C T G | 1e-25 | -5.940e+01 | 2.99% | 0.82% | 54.9bp (66.7bp) | Tcf4(HMG)/Hct116-Tcf4-ChIP-Seq(SRA012054)/Homer(0.786) More Information \| Similar Motifs Found | motif file (matrix) |
| 26 | C T A G A C T G T A C G A T C G A T C G T A C G A C T G | 1e-24 | -5.659e+01 | 30.09% | 22.44% | 55.8bp (64.8bp) | ZNF740/MA0753.1/Jaspar(0.919) More Information \| Similar Motifs Found | motif file (matrix) |
| 27 | A T C G C G A T A C T G A C T G T A C G C G T A C T G A A C T G C G A T T C A G T C G A A C G T | 1e-24 | -5.653e+01 | 1.51% | 0.20% | 53.1bp (75.2bp) | ABF1/ABF1\_YPD/62-ABF1(Harbison)/Yeast(0.688) More Information \| Similar Motifs Found | motif file (matrix) |
| 28 | G T A C C A G T C A T G A G C T G A T C G C T A G A T C G T C A G A C T A T G C | 1e-23 | -5.328e+01 | 6.90% | 3.35% | 53.8bp (65.5bp) | Hth/dmmpmm(Noyes\_hd)/fly(0.796) More Information \| Similar Motifs Found | motif file (matrix) |
| 29 | C G A T C T G A T C A G A G T C C G T A T A G C A G T C A C G T A G T C A G T C A G T C | 1e-23 | -5.315e+01 | 2.02% | 0.42% | 45.7bp (60.6bp) | Tbx5(T-box)/HL1-Tbx5.biotin-ChIP-Seq(GSE21529)/Homer(0.698) More Information \| Similar Motifs Found | motif file (matrix) |
| 30 | T C G A T C A G A G T C C T A G A C T G T G C A G T C A | 1e-22 | -5.270e+01 | 16.76% | 11.04% | 53.8bp (67.5bp) | RDR1/MA0360.1/Jaspar(0.893) More Information \| Similar Motifs Found | motif file (matrix) |
| 31 | T C G A G T A C A T G C C T G A A T G C G C T A T G C A A T C G C T G A A C T G | 1e-22 | -5.181e+01 | 3.07% | 0.96% | 50.6bp (64.9bp) | UNC-75(RRM)/Caenorhabditis\_elegans-RNCMPT00081-PBM/HughesRNA(0.714) More Information \| Similar Motifs Found | motif file (matrix) |
| 32 | A C T G A T G C C G A T A C T G C T A G T A G C C G T A A C T G A C T G T G C A C T G A T A C G T A G C | 1e-22 | -5.143e+01 | 2.91% | 0.88% | 46.3bp (72.6bp) | SRSF9(RRM)/Homo\_sapiens-RNCMPT00067-PBM/HughesRNA(0.697) More Information \| Similar Motifs Found | motif file (matrix) |
| 33 | A C T G C T G A A C T G C G T A C G T A A T G C A C G T | 1e-21 | -5.035e+01 | 12.90% | 8.00% | 53.3bp (70.5bp) | LIN28A(CSD,Znf)/Homo\_sapiens-RNCMPT00036-PBM/HughesRNA(0.777) More Information \| Similar Motifs Found | motif file (matrix) |
| 34 | C A T G A G T C A T C G A C T G G A C T G C T A C T G A C T G A | 1e-20 | -4.682e+01 | 21.58% | 15.49% | 57.3bp (63.2bp) | GTL1(Trihelix)/colamp-GTL1-DAP-Seq(GSE60143)/Homer(0.834) More Information \| Similar Motifs Found | motif file (matrix) |
| 35 | A T G C C A T G A C G T A C G T A C G T T C G A A G T C | 1e-19 | -4.384e+01 | 24.35% | 18.14% | 58.1bp (65.0bp) | GTL1(Trihelix)/colamp-GTL1-DAP-Seq(GSE60143)/Homer(0.827) More Information \| Similar Motifs Found | motif file (matrix) |
| 36 | A C T G T A C G A G C T A C G T C G A T C A T G T G A C C T A G C T A G | 1e-18 | -4.273e+01 | 18.97% | 13.47% | 55.9bp (63.9bp) | CG7903(RRM)/Drosophila\_melanogaster-RNCMPT00144-PBM/HughesRNA(0.781) More Information \| Similar Motifs Found | motif file (matrix) |
| 37 | A T G C G C T A T A C G G T A C G A C T A C T G A T G C G C A T A C T G A T G C G A T C | 1e-18 | -4.253e+01 | 10.53% | 6.45% | 57.2bp (64.5bp) | MyoD(bHLH)/Myotube-MyoD-ChIP-Seq(GSE21614)/Homer(0.837) More Information \| Similar Motifs Found | motif file (matrix) |
| 38 | C G A T A G T C A C G T G A C T A G C T A G C T C G T A T C G A T G C A A T G C G C T A C T G A G A T C G T A C | 1e-18 | -4.166e+01 | 1.02% | 0.12% | 54.3bp (63.4bp) | KHDRBS1(KH)/Mus\_musculus-RNCMPT00062-PBM/HughesRNA(0.645) More Information \| Similar Motifs Found | motif file (matrix) |
| 39 | C T G A A G T C A G C T A C G T A G T C A G T C G C A T A T G C A G C T A G T C G T A C A G T C C G A T | 1e-17 | -4.111e+01 | 1.08% | 0.14% | 55.0bp (67.1bp) | FRS9(ND)/col-FRS9-DAP-Seq(GSE60143)/Homer(0.725) More Information \| Similar Motifs Found | motif file (matrix) |
| 40 | C G T A C G T A G T C A A G T C C T G A A C T G A T C G C G T A C T G A C G T A G T A C A T C G | 1e-17 | -4.070e+01 | 0.46% | 0.00% | 32.5bp (2.2bp) | EWS:FLI1-fusion(ETS)/SK\_N\_MC-EWS:FLI1-ChIP-Seq(SRA014231)/Homer(0.768) More Information \| Similar Motifs Found | motif file (matrix) |
| 41 | C T A G G A T C C A T G A T C G A C G T A C G T A G T C G A T C A C G T A G C T | 1e-17 | -3.999e+01 | 2.07% | 0.59% | 35.0bp (66.0bp) | OPI1/MA0349.1/Jaspar(0.723) More Information \| Similar Motifs Found | motif file (matrix) |
| 42 | C G T A A C T G T A C G G T A C C G A T A C T G A C G T A C G T C T G A | 1e-17 | -3.963e+01 | 20.29% | 14.83% | 58.7bp (64.8bp) | MYB(HTH)/ERMYB-Myb-ChIPSeq(GSE22095)/Homer(0.753) More Information \| Similar Motifs Found | motif file (matrix) |
| 43 | G A C T C G A T A T C G C A G T A C T G A G T C C G A T T A C G C G T A C A T G A C G T T A G C G C T A G A C T | 1e-17 | -3.957e+01 | 1.72% | 0.42% | 57.7bp (67.5bp) | Bach1(bZIP)/K562-Bach1-ChIP-Seq(GSE31477)/Homer(0.905) More Information \| Similar Motifs Found | motif file (matrix) |
| 44 | G C A T A C G T G A T C G T C A A C G T G A T C A G C T T C G A G T C A G T A C A G T C G T C A A G T C G T C A | 1e-17 | -3.954e+01 | 0.65% | 0.03% | 56.0bp (74.9bp) | RUNX(Runt)/HPC7-Runx1-ChIP-Seq(GSE22178)/Homer(0.735) More Information \| Similar Motifs Found | motif file (matrix) |
| 45 | C G T A A G C T T A G C C G T A T A C G G T C A C A G T | 1e-16 | -3.881e+01 | 27.94% | 21.76% | 56.6bp (64.2bp) | CG14718(RRM)/Drosophila\_melanogaster-RNCMPT00006-PBM/HughesRNA(0.804) More Information \| Similar Motifs Found | motif file (matrix) |
| 46 | C G A T A C T G C G T A A C G T C G T A G C T A C T G A T C G A C G T A G A C T A G T C C G T A | 1e-16 | -3.836e+01 | 0.51% | 0.01% | 61.1bp (35.4bp) | GATA(Zf),IR4/iTreg-Gata3-ChIP-Seq(GSE20898)/Homer(0.760) More Information \| Similar Motifs Found | motif file (matrix) |
| 47 | A C G T T A G C A G C T A T G C C T G A G A C T C T A G C T G A A C T G C T G A G C T A G A T C A C T G | 1e-16 | -3.817e+01 | 2.67% | 0.94% | 51.8bp (64.4bp) | Su(H)/dmmpmm(Bergman)/fly(0.712) More Information \| Similar Motifs Found | motif file (matrix) |
| 48 | C G T A C G T A C G T A C G T A A C T G A C T G A C T G C G T A | 1e-16 | -3.721e+01 | 3.93% | 1.75% | 44.2bp (73.1bp) | CDF3(C2C2dof)/colamp-CDF3-DAP-Seq(GSE60143)/Homer(0.833) More Information \| Similar Motifs Found | motif file (matrix) |
| 49 | A T C G C T G A T C A G T A G C T C G A A C T G T A C G C T G A T A C G T G A C T C G A C T A G T C A G T C G A | 1e-15 | -3.575e+01 | 4.47% | 2.15% | 59.2bp (77.5bp) | ZNF263/MA0528.1/Jaspar(0.664) More Information \| Similar Motifs Found | motif file (matrix) |
| 50 | A C G T C T A G C G A T G T A C C G T A G C A T C T G A | 1e-15 | -3.468e+01 | 32.89% | 26.72% | 58.4bp (62.9bp) | Achi/dmmpmm(Noyes\_hd)/fly(0.882) More Information \| Similar Motifs Found | motif file (matrix) |
| 51 | A G T C A G C T A C T G A G T C A G C T A G C T G T A C G T A C A T C G C G A T A C G T A C G T | 1e-15 | -3.460e+01 | 2.75% | 1.05% | 58.2bp (66.8bp) | Adf1/dmmpmm(Bergman)/fly(0.659) More Information \| Similar Motifs Found | motif file (matrix) |
| 52 | C T G A G T A C C G T A A C T G T C A G G T C A C G T A A C T G T C A G G T C A A C G T A T C G | 1e-14 | -3.287e+01 | 0.67% | 0.06% | 47.9bp (72.6bp) | ERG(ETS)/VCaP-ERG-ChIP-Seq(GSE14097)/Homer(0.730) More Information \| Similar Motifs Found | motif file (matrix) |
| 53 | A C G T A G T C C G T A A G T C C G T A C A T G A C T G A G T C A C G T C T A G A G C T G T A C G T C A | 1e-14 | -3.274e+01 | 1.00% | 0.17% | 53.8bp (57.4bp) | HAC1/Literature(Harbison)/Yeast(0.725) More Information \| Similar Motifs Found | motif file (matrix) |
| 54 | C A T G A G C T A C G T A G T C C G A T G A T C G C T A G A T C T C A G G T C A | 1e-13 | -3.214e+01 | 2.26% | 0.80% | 52.7bp (68.6bp) | Su(H)/dmmpmm(Papatsenko)/fly(0.828) More Information \| Similar Motifs Found | motif file (matrix) |
| 55 | C T A G C T A G A C T G A C T G T C G A C T G A C T A G C T G A C T A G A C T G A C T G C T A G A C T G | 1e-13 | -3.196e+01 | 2.13% | 0.73% | 50.9bp (71.8bp) | Zfp281(Zf)/ES-Zfp281-ChIP-Seq(GSE81042)/Homer(0.681) More Information \| Similar Motifs Found | motif file (matrix) |
| 56 | C T A G T G A C A G C T A C G T C T A G C T G A A G T C A C G T A C G T A T G C G A C T | 1e-13 | -3.146e+01 | 1.24% | 0.28% | 49.4bp (66.8bp) | Nr2e1/MA0676.1/Jaspar(0.815) More Information \| Similar Motifs Found | motif file (matrix) |
| 57 | A T C G C T G A A C T G A T C G T G C A C T G A C T A G A T C G G T C A T C G A T G C A A T G C G T A C | 1e-13 | -3.114e+01 | 1.21% | 0.27% | 44.7bp (67.9bp) | RBM5(Znf)/Homo\_sapiens-RNCMPT00055-PBM/HughesRNA(0.758) More Information \| Similar Motifs Found | motif file (matrix) |
| 58 | T C A G A G T C C T G A A G T C T A G C C G A T A T G C A T C G G A C T | 1e-13 | -3.073e+01 | 7.30% | 4.42% | 58.1bp (64.8bp) | RCS1(MacIsaac)/Yeast(0.674) More Information \| Similar Motifs Found | motif file (matrix) |
| 59 | C G T A C T G A A C G T A C T G C T G A A C T G T G C A C G T A T A G C C G A T A C T G T A G C A G T C G A C T | 1e-13 | -3.023e+01 | 0.46% | 0.02% | 60.8bp (47.8bp) | PB0099.1\_Zfp691\_1/Jaspar(0.660) More Information \| Similar Motifs Found | motif file (matrix) |
| 60 | G C T A A C G T G T A C G A C T C A T G A C T G A G C T C G A T C T A G A G C T C G A T | 1e-13 | -3.017e+01 | 1.27% | 0.30% | 50.7bp (63.2bp) | grh/dmmpmm(Bigfoot)/fly(0.698) More Information \| Similar Motifs Found | motif file (matrix) |
| 61 | T A C G A G T C A C G T A C T G A G T C T G C A A T C G A T G C C G A T A C T G A G T C G T A C G C T A T A G C | 1e-12 | -2.904e+01 | 0.59% | 0.05% | 57.5bp (57.8bp) | Myog/MA0500.1/Jaspar(0.752) More Information \| Similar Motifs Found | motif file (matrix) |
| 62 | G T A C G A C T A C G T A G T C C G A T A T C G G A T C G A C T G C T A A C G T A C T G G C T A C A T G G C A T | 1e-12 | -2.880e+01 | 1.00% | 0.20% | 54.7bp (59.2bp) | REF2(RRM)/Drosophila\_melanogaster-RNCMPT00059-PBM/HughesRNA(0.678) More Information \| Similar Motifs Found | motif file (matrix) |
| 63 | A T C G G C A T G C A T C T G A T A C G G T A C C G A T T C A G G A T C G A C T | 1e-12 | -2.868e+01 | 15.27% | 11.19% | 57.2bp (65.8bp) | grh/dmmpmm(Papatsenko)/fly(0.702) More Information \| Similar Motifs Found | motif file (matrix) |
| 64 | G T A C A C T G A C G T C T G A C T G A C G T A C G T A C A G T A T C G | 1e-12 | -2.817e+01 | 3.93% | 1.99% | 59.7bp (62.5bp) | Lm\_0212(RRM)/Leishmania\_major-RNCMPT00212-PBM/HughesRNA(0.854) More Information \| Similar Motifs Found | motif file (matrix) |
